# Organic Amendments Effects on Nutrient Uptake, Secondary Metabolites, and Antioxidant Properties of *Melastoma malabathricum* L.

**DOI:** 10.3390/plants11020153

**Published:** 2022-01-06

**Authors:** Lili Syahani Rusli, Rosazlin Abdullah, Jamilah Syafawati Yaacob, Normaniza Osman

**Affiliations:** 1 Institute of Biological Sciences, Faculty of Science, Universiti Malaya, Kuala Lumpur 50603, Malaysia; lilis369@uitm.edu.my (L.S.R.); rosazlin@um.edu.my (R.A.); 2 Faculty of Applied Sciences, Universiti Teknologi MARA, Cawangan Negeri Sembilan, Kampus Kuala Pilah, Kuala Pilah 72000, Malaysia; 3 Centre for Research in Biotechnology for Agriculture (CEBAR), Universiti Malaya, Kuala Lumpur 50603, Malaysia

**Keywords:** *Melastoma malabathricum* L., palm kernel biochar, food waste compost, soil fertility, plant phytochemical

## Abstract

Amelioration of soil acidity can boost soil fertility, hence increasing nutrient uptake, secondary metabolite, and its antioxidant potential. In the present study, the effectiveness of food waste compost and palm kernel biochar was assessed as soil amendments for *Melastoma malabathricum* L. grown in acidic soil conditions. A six-month greenhouse study was conducted using completely randomized design (CRD) with three treatment groups, including control plants (T1), plants amended with palm kernel biochar (T2), and plants amended with food waste compost (T3). Data analysis revealed that *Melastoma malabathricum* L. amended with T3 recorded the highest total chlorophyll content (433.678 ± 13.224 µg g^−1^ DW), followed by T2 and T1. The increase in chlorophyll content was contributed by the increase in soil pH. This was shown by the positive significant correlations between soil pH and chlorophyll a (r^2^ = 0.96; *p* ≤ 0.01) and chlorophyll b (r^2^ = 0.778; *p* ≤ 0.01). In addition, the same treatment exhibited the highest total anthocyanin content (leaves; 36.1 × 10^−2^ ± 0.034 mg/g DW and root extract; 8.9 × 10^−2^ ± 0.020 mg/g DW), total phenolic content (stem extract; 4930.956 ± 16.025 mg GAE/g DE), and total flavonoid content (stem extract; 209.984 ± 0.572 mg QE/g DE). Moreover, this study also found that the highest antioxidant potential against 2,2-Diphenyl-1-picrylhydrazyl (DPPH) and 2,2-Azinobis (3-ethylbenzothiazoline-6-sulfonic acid (ABTS) radicals was exhibited by samples supplemented with food waste compost (T3), followed by palm kernel biochar (T2). This indicates that the soil amendments have the capacity to enhance the secondary metabolites that protect plants, therefore ameliorating *Melastoma malabathricum* L.’s response towards acidic stress, and resulting in better antioxidant properties. Furthermore, this study also recorded better nutrient uptake in T3. With the significantly higher levels of macronutrient in the soil, the food waste compost could enhance the nutrient properties, secondary metabolites, and antioxidant capacity of *Melastoma malabathricum* L. grown in acidic soil conditions.

## 1. Introduction

Organic soil amendments are among the sustainable management practices used to enhance soil fertility and have been proven to positively improve poor soils’ nutrient content and other soil chemical properties [1,2]. Organic soil amendments such as compost and biochar have been shown to enhance soil function by increasing the water-holding capacity, porosity, and surface area [3]. For example, a study by Visconti et al. (2020) reported a maximum of two-fold increment of total nitrogen and total organic carbon in soils treated with compost and biochar. They also reported that biochar produced from residues of orchard pruning reduced Zn, Cd, and Pb concentrations (32%, 8%, and 58%, respectively) along with compost from olive mill waste (45%, 20%, and 63%, respectively) compared to control treatment [4]. The benefits of organic soil amendments outweigh the disadvantages, and the benefits of recycling waste such as municipal and agricultural waste further make them a cost-effective yet environmentally friendly alternative to inorganic fertilizers. Composting is an economical, simplified, and cost-effective technique of managing solid organic waste that maximizes nutrient recycling [5]. Local composting from household wastes is regarded as a sustainable method that is gaining great demand. The major composition of household waste in South East Asia is food waste, followed by paper, plastic, metal, and glass [6]. Composting food waste (FW) is one of the most effective methods for treating biodegradable waste components; it is also one of the potential waste management aspects for diverting trash generated from landfills while recycling organic materials through conversion into a valuable product. Food waste compost, in addition, has been proven to increase the concentration of soil nutrients such as soil organic matter, dissolving organic carbon, total nitrogen, soil ammonium (NH_4_^+^), and nitrate NO_3_^−^) [7,8]. However, according to Van Fan et al. (2018), FW compost has some limitations such as low C/N, high moisture content, and low porosity depending on the quality of the initial materials in the compost [9].

In addition, biochar, the byproduct of biomass and organic waste through thermal degradation [10] has been reported to enhance crop yields and mitigate global warming [11], significantly reducing soil N_2_O emissions, and increasing the total soil organic carbon content [12]. When applied to soil, biochar can help reduce the bulk density of the soil and increase its overall porosity, water retention capacity, and cation exchange capacity [13,14]. In addition, biochar as a soil amendment can increase the soil nutrient content and crop production [15]. It has also been reported that biochar from oil palm waste has ameliorative properties, including the ability to absorb soil pollutants and decrease the acidity of soil [16]. Due to the promising features, researchers have started to examine the use of palm kernel (PK) biochar as a growth medium in ornamental plants, *Coleus* sp. which demonstrated superior performance in terms of plant growth, physiological performance, and total nitrogen and organic matter content [17].

The range of soil pH in Malaysia is 3.04 to 7.01 with a mean of 5.40 [18], indicating that acid soil occupies most of the land areas in the country. Soil acidification has been reported to cause toxic heavy metal accumulation, affected the biotic community distribution, and disturbed nutrient absorption in soil [19,20,21]. Due to these factors, soil acidity therefore limits plant growth and decrease the yield of various crop species [22]. Nevertheless, there has been little research on the mechanisms of acid tolerance in soil-grown plants [20,23]. However, it has been postulated that the growth of plants on acidic soil is reduced due to Al or Mn toxicities [24], and P, Ca, and Mg deficiencies [25].

*Melastoma malabathricum* L., commonly known as sendudok, is an invasive weed species in the Malaysian agrosystem that belongs to the family of Melastomataceae. This shrub plant is one of the natural plant resources that has gained attention within the scientific world due to its ethnomedicinal values. This plant has also been reported to be growing wild in the Indian Ocean Islands, and throughout South and South-East Asia, China, Taiwan, Australia, and the South Pacific Ocean [26]. *M. malabathricum* L. can grow as small shrubs up to 3 ft in height, occasionally even up until 15 ft [27]. Traditional medicine practitioners commonly use this species as a remedy for diarrhea, gastric ulcers, inflamed wounds, pox scars, diabetes, and high blood pressure [28,29]. In addition, numerous secondary metabolites have been discovered in *M. malabathricum* L., such as anthocyanin [30], flavonoids, and phenolic contents [31]. Owing to its ability to tolerate harsh acidic conditions, this plant has been known to be a potential Al accumulator [32,33]. The M-type roots also enable the plants to grow extensively in various directions and are beneficial in controlling soil erosion by improving the mechanical and hydrological properties [34], thus making them a potential slope remediator plant species [35]. Various studies have been conducted on plants to study the effect of soil amendments on the soil physicochemical, bioactive compound, and antioxidant activities on individual crops; however, little is known about those effects on *M. malabathricum* L. grown in acidic conditions. Currently, our understanding on the effects of various organic amendments on *M. malabathricum* L. is limited. No study investigates the effect of palm kernel biochar and food waste compost on the nutrient uptake, phytochemical profiles and antioxidative properties of *M. malabathricum* L. under acidic stress was undertaken. A thorough study on the effect of organic amendments on nutrient uptake, secondary metabolites, and antioxidant activity of *M. malabathricum* L. is crucial for maximizing the benefits of this plant. Thus, to fill the knowledge gap, this study investigates whether the nutrients adsorbed by the organic amendments used could be available to support *M. malabathricum* L.’s growth in acidic soil conditions. Additionally, this study was conducted with the objective of examining the effects of FW compost and PK biochar on the nutrient uptake, secondary metabolites, and antioxidant potential of *M. malabathricum* L.

## 2. Results

### 2.1. Soil Analysis and Mineral Content

**Figure 1:**
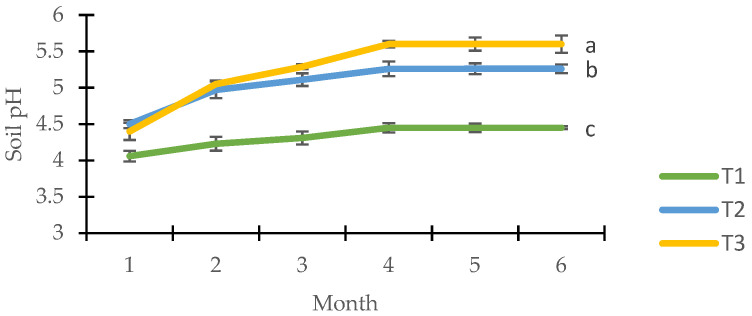
Effects of different amendments on soil pH (*n* = 3). Vertical bars represent the standard deviation. Different letters indicate a significant difference (*p* < 0.05) amongst treatments in the final planting period.

Soil amendments increased the soil pH from 3.9 to 5.6 (Figure 1). Significantly higher soil pH was observed in T3 (5.64 ± 0.069), followed by T2 (5.27 ± 0.035), and T1 (4.45 ± 0.02) at the final planting stage. Soil pH in T3 increased to 27.2% from the initial planting, followed by T2 and T1 with 15.5% and 9.6%, respectively. Evidently, T1 took longer to completely disintegrate and react with the soil to maintain its pH level.

**Figure 2:**
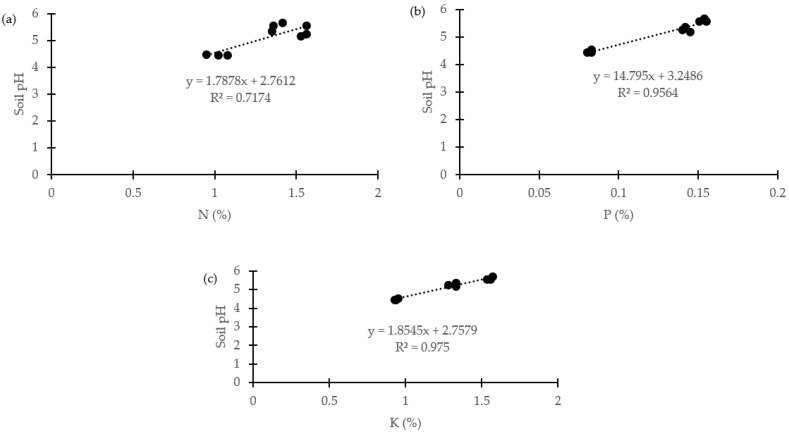
Relationship between soil pH and plant macronutrients with (**a**) N: nitrogen, (**b**) P: phosphorus, and (**c**) K: potassium (data are means of treatments).

The regression analysis showed a significant positive linear relationship between pH and plant macronutrients N (Figure 2a) with r^2^ = 0.717. Both P and K macronutrients exhibited strong positive linear relationships with r^2^ = 0.956 and 0.975, respectively (Figure 2b,c). This clearly shows that soil amendments increased the nutrient concentrations in the plants.

After harvest, the chemical characteristics of acidic soil were affected by the soil amendment applications as shown in Table 1. Significantly higher levels of macronutrients N (12.3 × 10^−2^ ± 0.009%), P (190.500 ± 1.500 mg/kg), and exchangeable cation of K (170 × 10^−2^ ± 0.151 meq/100 g), Ca (2.280 ± 0.142 meq/100 g), Mg (27.7 × 10^−2^ ± 0.020 meq/100 g), and Na (4.0 × 10^−2^ ± 0.006 meq/100 g) were reported in the T3 treatment, whereas the control had the highest levels of exchangeable micronutrients (Zn (2.653 ± 0.018 mg/kg), Fe (191.270 ± 5.047 mg/kg), Mn (2.653 ± 0.034 mg/kg), Cu (31.090 ± 0.517 mg/kg), Cd (79.3 × 10^−2^ ± 0.054 mg/kg), and Al (2.1 × 10^−2^ ± 0.001 mg/kg)). Exchangeable Ca and Na in the T3 treatment were also found to be comparable with those in the T2 treatment. This study shows that exchangeable Zn, Fe, Mn, and Cu in the soils can be significantly reduced by applying soil amendments. Meanwhile, the highest exchangeable Cd and Al were reported in the T3 treatment.

### 2.2. Phytochemical Screening

The methanolic extracts from different plant parts of *M. malabathricum* L. were analyzed and data analysis revealed the presence of flavonoid and phenols in all samples (Table 2). However, no presence of phlobatannins and saponins were detected in all samples. Meanwhile, tannins were observed to be present in the leaves and root extract, while alkaloid was only present in the leaves extract.

### 2.3. Determination of Total Chlorophyll and Carotenoid Content

The values for chlorophyll a (C*a*) and b (C*b*) showed similar trends (Table 3). T3 exhibited the highest value for C*a* (289.441 ± 8.881 µg g^−1^ DW) with 10.54% and 58.49% higher than T2 and T1, respectively. Significantly higher values of C*b* (144.237 ± 4.585 µg g^−1^ DW) C*a* + C*b* (433.678 ± 13.224 µg g^−1^ DW), and carotenoid content (237.733 ± 7.224 µg g^−1^ DW) were also obtained in T3, followed by T2 and T1. A significantly higher C*a*/C*b* ratio (2.756 ± 0.011) was recorded in T2; however, no significant difference could be observed between T1 and T3. The highest value of C*a* + C*b/C(x+c)* ratio recorded in T1 was 1.836 ± 0.028, followed by T3 and T2 with C*a* + C*b/C(x+c)* ratios of 1.824 ± 0.014 and 1.588 ± 0.006, respectively.

### 2.4. Determination of Total Anthocyanin, Flavonoid and Phenolic Contents

The total anthocyanin (TAC), total phenolic (TPC), and total flavonoid (TFC) contents in the methanolic extracts of the various parts of *M. malabathricum* L. were measured. The TPC was expressed as mg gallic acid (GAE) per g dry extract, whereas the TFC was expressed as mg quercetin (QE) per g dry extract. Based on Table 4, in the leaf samples, the highest TAC was obtained in T3 (36.1 × 10^−2^ ± 0.034 mg/g DW), followed by T2 and T1. The data analysis further revealed that the differences observed in both T3 and T2 were statistically significant compared to T1. The root also exhibited the highest TAC in T3 (8.9 × 10^−2^ ± 0.020 mg/g DW), followed by T2 and T1. However, the TAC was observed to be the highest in T2 (6.1 × 10^−2^ ± 0.024 mg/g DW), followed by T3, and the lowest in T1 in the stem of *M. malabathricum* L., while all samples in the root and stem were not statistically significant in terms of TAC.

The TPC was observed to be significantly higher for T3 in the stem (4930.956 ± 16.025 mg GAE/g DE) compared to T2 (2267.808 ± 58.939 mg GAE/g DE) and T1 (2174.517 ± 27.789 mg GAE/g DE). Significantly higher TPC was also found in the methanolic extracts of T2 (5419.291 ± 36.121 mg GAE/g DE) and T3 (5396.671 ± 8.200 mg GAE/g DE) roots, compared to T1 (3606.996 ± 9.396 mg GAE/g DE). T2 (9933.322 ± 30.217 mg GAE/g DE) was also observed to comprise the highest TPC, followed by T3 (9857.329 ± 49.172 mg GAE/g DE) and T1 (9505.160 ± 182.057 mg GAE/g DE) in the leaves. The same trend was observed for the TFC, where the highest TFC was obtained by T3 in stem and T2 in leaves and root, respectively.

### 2.5. Antioxidant Potential of M. malabathricum L. methanolic Extracts

The antioxidant activities expressed by ABTS and DPPH assays of the *M. malabathricum* L. methanolic extracts are displayed in Table 5. The antioxidant activity of the leaf extracts against both ABTS and DPPH radicals, in increasing order, is T1 < T2 < T3. Analysis of the data showed that the leaves’ methanolic extract in T3 exhibited significantly highest radical scavenging activity (denoted by the lowest IC_50_) against ABTS and DPPH with an IC_50_ value of 27.9 × 10^−2^ ± 0.020 mg/mL and 13.1 × 10^−2^ ± 0.001 mg/mL, respectively, compared to control, T1. A similar trend was observed in the antioxidant activity of the root extract. However, the data analysis showed that the T2 stem methanolic extract recorded the lowest IC_50_ values, with 16.5 × 10^−2^ ± 0.002 mg/mL (ABTS) and 73.4 × 10^−2^ ± 0.039 mg/mL (DPPH), followed by T3.

### 2.6. Significant Pearson’s Correlation between Measured Parameters

Pearson’s correlation analysis was carried out to determine the relationships among the soil nutrient content, secondary metabolites, and antioxidant potential (Table 6). Analysis of the data revealed a strong positive correlation between soil pH and N (r^2^ = 0.929). Soil pH also demonstrated strong positive correlations with P (r^2^ = 0.938), K (r^2^ = 0.944), total chlorophyll content (r^2^ = 0.950), carotenoid content (r^2^ = 0.945), TAC (r^2^ = 0.869), and TFC (r^2^ = 0.883) with *p* ≤ 0.01, respectively. This indicates that soil macronutrients, total chlorophyll content, carotenoid content, TAC, and TFC would increase significantly with the increase in soil pH. In contrast, strong negative correlations between soil pH and Zn (r^2^ = −0.802), Cu (r^2^ = −0.940), DPPH (r^2^ = −0.979), and ABTS (r^2^ = −0.822) were respectively observed. Soil macromolecules (N, P, K) showed strong positive correlations with total chlorophyll, but a strong negative correlation with DPPH. Similar trend was also observed in C*a*, C*b*, C*a* + C*b* and carotenoid which exhibited a strong negative correlation with DPPH and ABTS (P ≤ 0.01). Both DPPH and ABTS resulted in a positive correlation with Zn and Cu, meanwhile the TPC, TFC, and TAC were negatively correlated with soil micronutrients such as Zn, Fe, and Mn.

## 3. Discussion

Based on the overall data analysis, soil amendments were found to influence the mineral composition of the soil. In reference to Figure 1, soil pH at the final planting stage was significantly influenced by the soil amendments, where the highest was recorded in T3, followed by T2 and T1 treatments. Hydrolysis occurs when the cation’s charge to size ratio is sufficiently large to disrupt the H-O bonds leads to hydrate ionization, which produces hydrogen ions that releases H^+^ and regenerates the Al^3+^ ions, permitting further reaction [36].

The presence of Al promotes the development of most plants acclimated to low pH soils in tropical and temperate locations, which is believed to be related to the increased N, P, and K uptake [37]. This explains the decrement of soil acidity up to 14% in *M. malabathricum* L. in acidic soil conditions during the final planting stage. This result is in agreement with the previous study, which has shown that Al accumulator plants adapted to low soil pH [37,38]. Furthermore, the decrease in soil acidification occurred as a result of the soil’s inherent Ca and Mg content being released. As the loss of basic cations by leaching is minimal, it is possible to increase soil pH by the accumulation of basic cations throughout the planting period [39].

Adding organic amendments (T3 and T2) significantly decreased soil acidification due to the higher pH of organic inputs from the amendments used (Figure 1). The elevated soil pH is a result of the rapid proton (H^+^) exchange between the soil and organic amendments [40]. The increase in soil pH also relates to the release of OH^−^ due to specific adsorption of humic material and organic acids produced by the compost and biochar onto the hydrous surfaces of Al and Fe oxides by ligand exchange [41]. In addition, further decomposition of the organic amendments was caused by further basic cation releases such as K, Ca, and Mg contents of the organic amendments to the soil [42]. In addition, food waste compost as soil amendments has also been reported to increase soil pH as a result of oxygen consumption by the chemical and/or biological processes [43]. By converting to anaerobic conditions, certain heterotrophic microorganisms may gain dominance by utilizing organic carbon as the source of food and energy [44].

Organic amendment treatments (T2 and T3) improved soil N by preventing NH_4_^+^ and NO_3_^−^ from being lost [45,46]. FW compost and PK biochar’s N content may have contributed to the rise in the total nitrogen in the soil. Soil N was significantly affected by the application of organic amendments (Table 1) and the findings indicated that both organic amendments increased soil nitrogen similarly. The highest percentage of soil N was recorded in the soil amended with FW compost, which was expected, given the fact that FW compost contained a substantially higher proportion of N than PK biochar. A study by Sierra (2001) examined the effect of leaching on the depletion of nutrients, particularly nitrogen, from the soil. Evidently, simultaneous leaching of sodium out of soil improves the circumstances for microbial activity; therefore, N was immobilized by moving to the microbial biomass triggered by organic matter decomposition [47].

Treatment with FW compost (T3) showed the highest amount of soil available P (Table 1). Additionally, the high affinity of functional groups in the inputs of organic soil amendments may have inhibited P fixation by Al and Fe [48]. The presence of more soil-available P in the FW compost treatment than in the PK biochar treatment indicated that compost is a more efficient fertilizer in terms of supplying available P nutrient [49], which may be associated with the increased microbial activity following the compost application and the P released during organic matter decomposition [50]. This is followed by the T2 treatment with PK biochar. Being more negatively charged, biochar readily binds to positively charged metal oxides such as Al_2_O_3_ and Fe_2_O_3_, a reaction that reduces the tendency of Al and Fe from reacting with soil-available P [48]. These findings are in agreement with the results that were reported by other studies on maize, which showed palm kernel biochar addition increased the available P by 13.9% in comparison to control [51].

Based on the organic amendments’ nutrient composition, organic amendments might be expected to have a fertilizing effect on soil exchangeable bases, K, Ca, and Mg. In this study, the observed increase in mineralization following soil amendment with FW compost could be attributed to the chemical composition used in FW compost, suggesting the chemical properties of the organic matter in the compost and the concentration of humic acids released into the soil, influencing the aggregate stability [52,53,54]. The improvement in soil productivity and fertility associated with the exogenous addition of organic matter will endure in the soil so long as the added organic matter is protected from microbial attack [52,55]. Basic cations, such as Ca^2+,^ Mg^2+^, and K^+^ in the form of oxides or carbonates can dissolve in water and produce OH^−^, which, in turn, increases the soil pH [56,57]. The carbonate content is responsible for the alkalinity of biochar [58,59,60] and was positively correlated with basic cation [61]. This could be attributed to the release of basic cations from both FW compost and PK biochar. High K content in PK biochar caused the reduced uptake of Ca in PK biochar (Table 1). Previous studies have corroborated this finding [62,63] by reporting a negative relationship between Ca and K. Based on this statement, the high input of K from PK biochar could be attributed to the low uptake of Ca in PK biochar.

Concerning potentially hazardous trace elements, which is the most researched risk in soil modified with urban waste, the components found in greater amounts in this study were Fe > Cu > Zn > Mn > Cd > Al (Table 1). The results confirmed the hypothesis that the use of FW compost and PK biochar as soil amendments improved the micronutrient contents. Our results indicated that the concentrations of Cu and Zn coincide with the concentration ranges for all Peninsular Malaysian topsoil [18]. However, Cd exceeded the maximum threshold level [18] and this result has also been reported by other authors [64] who investigated the leaching of metals in relation to the application of metal immobilizing soil amendments, suggesting that the destruction of soil aggregates has an effect on (de)sorption processes, and oxidation of soil functional groups alters the structure of compounds and their ability to bind metals, resulting in increased metal mobility.

The positive linear relationship between pH and plant macronutrients (Figure 2) clearly indicates that soil treated with organic amendments improved plant nutrient uptake. This further indicates that the acidic soil becomes fertile with the external input from organic amendments [65].

The qualitative screening of Melastomataceae leaf extracts has been widely reported [66,67,68]; however, to date, no available studies have reported on the biochemical properties of various parts of *M. malabathricum* L supplemented with FW compost and PK biochar. The results revealed the presence of flavonoids and phenols (Table 2). A similar phytochemical was also reported to be present in the leaves of *M. malabathricum* L. [29,69]. Meanwhile, tannins were observed to be present in the leaf and root extracts, while alkaloid was present only in the leaf extract.

*M. malabathricum* L. leaf pigment contents were significantly influenced by different soil amendments (Table 3). This study has shown that FW compost significantly increased the release of carotenoid in the leaves along with the enhancement of chlorophyll content, and this observation was supported by the strong positive correlations between the carotenoid content with chlorophyll a (r^2^ = 0.994, *p* ≤ 0.01), chlorophyll b (r^2^ = 0.687 *p* ≤ 0.05), and total chlorophyll (r^2^ = 0.940 *p* ≤ 0.01 (Table 6). Similar findings were observed in a study by Neagoe (2005), which reported that the highest chlorophyll and carotenoid content were produced when rye (*Secale cereale* L.) and lupine (*Lupinus angustifolius* L.) were grown with municipal compost under acid mine drainage [70]. The application of FW compost significantly improved the leaf enzymatic activities, which can alleviate the reactive oxygen species (ROS) stress [71] caused by acidification. Hence, this led to chlorophyll and carotenoid content increase with the application of FW compost. The increased chlorophyll and carotenoid content in the leaves may also be associated with the increased nitrogen availability in FW compost and PK biochar amendment with improved water balance [72]. This is parallel to the results in this study where soil N had a significant positive correlation with total chlorophyll and carotenoid with 0.851 and 0.914, respectively, at a *p*-value of less than 0.01 (Table 7).

Organic amendments were found to increase the production of the total anthocyanin content (TAC), total phenolic content (TPC), and total flavonoid content (TFC) in *M. malabathricum* L. leaves grown in acidic soil conditions. The leaf methanolic extracts obtained from FW compost contained the highest TAC, followed by PK biochar and control plants (Table 4). A similar trend was observed in the root methanolic extracts. Both TPC and TFC of the leaf and root extracts exhibited a similar trend, with the highest TPC was recorded in PK biochar, followed by FW compost, and the lowest was exhibited by the control plants. On the contrary, Yusof et al. (2018) reported that with the lower TAC, TPC, and TFC values, organic amendment (vermicompost) had no significant effect on the expression of bioactive compounds [73]. They argued that Al toxicity could result in a rise in ROS, which could either boost or decrease antioxidant ROS-scavenging activities. However, such a condition was not observed in the current study. This shows that, when *M. malabathricum* L was supplemented with organic amendments, the plant or plant cells experience elicitation or enhanced biosynthesis of secondary metabolites due to the addition of trace amounts of elicitors [74]. Elicitors act to stimulate a response in plants from biotic or abiotic sources, resulting in increased synthesis and accumulation of secondary metabolites or the induction of novel secondary metabolites [75], as observed in this study with FW compost and PK biochar supplementations. In addition, *M. malabathricum* L. has been proven to contain higher anthocyanin accumulation with pH from 5.25 to 6.25 [76], achieved with the supplementation of organic amendments, especially FW compost, and annotated by a high positive correlation between TAC with pH (r^2^ = 0.869, *p* ≤ 0.01) (Table 6).

Based on the results, the leaf, stem, and root methanolic extracts of *M. malabathricum* L. plants supplemented with FW compost showed better DPPH and ABTS radical scavenging activities, followed by PK biochar and control plants. These findings are in agreement with that reported in the study by Lakhdar et al. (2011), which showed that the DPPH radical scavenging activity of *Mesembryanthemum edule* supplied with municipal solid waste compost had improved by 44% [77]. They further suggested that the high scavenging activity was due to the presence of hydroxyl groups in the phenolic compounds’ chemical structure that provides the necessary component as a radical scavenger. As the concentrations of P and K increased, the IC_50_ value of *M. malabathricum* L. was reduced, leading to its better scavenging activity (Table 5). The addition of FW compost and PK biochar also caused P and K to accumulate in the soil, hence its availability to be used by plants. In addition, the high P concentration in compost-amended soil bounded by clay minerals and organic matter becomes more available as a result of the action of organic acids released during decomposition [78,79].

## 4. Materials and Methods

### 4.1. Experimental Design and Sample Preparation

The study was carried out based on Complete Randomized Design with three treatments and six replications at a glasshouse located at Rimba Ilmu, Institute of Biological Sciences, Universiti Malaya, Kuala Lumpur, Malaysia (3°7′52.1076″ N, 101°39′25.218″ E) from February 2020 to July 2020. The experimental treatments comprised of *M. malabathricum* L. as control plants (T1), *M. malabathricum* L. supplied with palm kernel biochar (T2), and *M. malabathricum* L. supplied with food waste compost (T3). *M. malabathricum* L. was identified by comparing the specimens in the Herbarium of the Forest Research Institute Malaysia (FRIM), Kepong, Selangor, which were deposited with the specimen voucher. The physical–chemical properties of the soils and the nutrient content of the organic fertilizers used in this study are listed in Table 7. The plants of each treatment were harvested in July 2020. Soil samples were collected, homogenized, air-dried, sieved using a 0.25 mm laboratory sieve for macro elements and trace elements and a 2.0 mm laboratory sieve for physical properties, and kept in an airtight container for further analysis.

**Figure 3:**
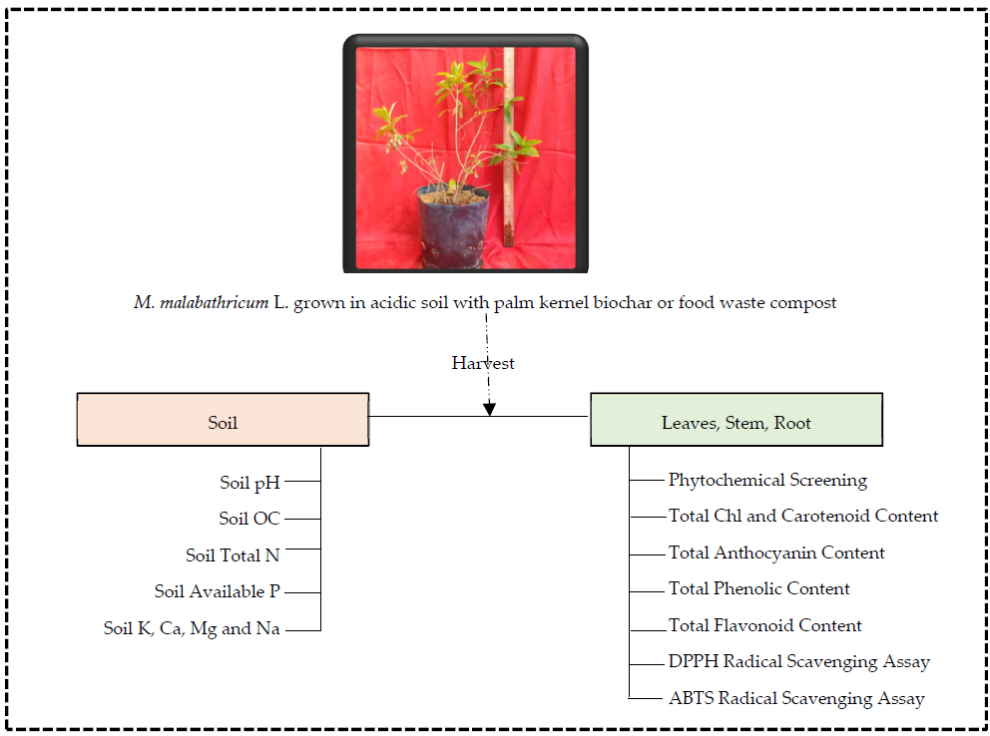
A schematic flow chart of methodology used in this study.

After harvesting, the methanolic extracts of *M. malabathricum* L. was prepared. The harvested plants were freeze-dried at −50 °C. A total of approximately 2.0 g of freeze-dried samples were ground in liquid nitrogen using chilled mortar and pestle. All extraction procedures were conducted in complete darkness. After homogenizing the samples, they were soaked in 60 mL of absolute methanol and incubated at 4 °C for 48 h. The filtrate was collected and stored at −20 °C. Re-extraction and filtration of the residue were performed and the extracts were combined and centrifuged for 5 min at 9000 rpm, 4 °C. A portion of these supernatants was immediately used for subsequent pigment analysis [80] to determine the total chlorophyll, carotenoid, and anthocyanin contents, while the remaining supernatants were concentrated to dryness using a rotavapor at 45 °C. The solvent-free extract was adjusted to a concentration of 10 mg/mL with absolute methanol and stored at −20 °C in an airtight glass vial until further analysis. The flow chart diagram of experimental outline is represented in Figure 3.

### 4.2. Soil Analysis

The soil pH was determined monthly, in a supernatant suspension of a 1:2.5 mixture of soil and distilled water [81]. The amount of soil organic carbon (OC) was estimated using the conversion factor of 1.724 following the assumption that organic matter includes 58% of organic carbon content [82,83]. The total nitrogen content of the soil was determined using the Kjeldahl distillation method [84], the Bray and Kurtz method for determining available P content [85], and the leaching method for determining K, Ca, Mg, and Na. The leachate was analyzed using Inductive Couple Plasma Optical Spectrometer [86].

### 4.3. Phytochemical Screening of Bioactive Compounds in M. malabathricum L.

The phytochemical screening was carried out on the methanolic extracts of *M. malabathricum* L. to detect the presence of bioactive compounds in the samples based on the standard protocols described by Solihah et al. [87].

### 4.4. Determination of Total Chlorophyll and Carotenoid Content

The methanolic extracts were analyzed in triplicate using a spectrophotometer (Multiskan^TM^ GO, Thermo Scientific, Waltham, MA, USA) at 652.4 and 665.2 nm. The concentrations of chlorophyll a, chlorophyll b, and carotenoid content were calculated based on the formula by Lichtenthaler and Buschmann [88]:

Chlorophyll a (µg/mL) = 16.72 A_665.2_ − 9.16 A_652_ (1)

Chlorophyll b (µg/mL) = 34.09 A_652.4_ − 15.28 A_665.2_ (2)

Chlorophyll (*x* + *c*) (µg/mL) = 1000 _A470_ − 1.63 *_Ca_* − 104.96 *_Cb_*/221 (3)

### 4.5. Measurement of Total Anthocyanin Content 

The total anthocyanin content (TAC) was measured using the pH differential method based on Giusti and Wrolstad (2001) [89] with some modifications. The samples were separately diluted with two types of buffer: potassium chloride (0.025 M) at pH 1.0 and sodium acetate (0.4 M) at pH 4.5 using the ratio of 1:4 (one-part test portion and four parts buffer) and the absorbance was read in triplicate at 520 and 700 nm using a spectrophotometer (Multiskan^TM^ GO, Thermo Scientific, Waltham, MA, USA. The anthocyanin pigment concentration was measured using the following formula:

(4) $$\mathrm{Anthocyanin}\;\mathrm{pigment}\;\mathrm{content}\;(\mathrm{mg}/L)=\frac{A\;\times \;\mathrm{MW}\;\times \;\mathrm{DF}\;\times \;1000}{\varepsilon \;\times \;1}$$

where A = (Abs_520_ − Abs_700_)pH1.0 − (Abs_520_ − Abs_700_)pH4.5 MW (Molecular weight of cyanidin − 3 − glucoside) = 449.2 g/mol DF = dilution factor *ε* = 26, 900

### 4.6. Measurement of Total Phenolic Content

The determination of the total phenolic content (TPC) of the methanolic extracts was conducted following the method described by Singleton et al. [90] with minor modifications. Briefly, 0.01 mL of the methanolic extracts was added with 0.75 mL of diluted Folin–Ciocalteu reagent (FCR) and incubated at room temperature for 10 min. Prior to incubation, FCR was diluted with deionized water. The mixture was then added with 0.75 mL of 2% Na_2_CO_3_ and further incubated for 45 min in the dark. The absorbance of the samples was read at 765 nm using a spectrophotometer (Multiskan^TM^ GO, Thermo Scientific, Waltham, MA, USA). Gallic acid with six different concentrations (ranging from 0.1 to 0.55 mg/mL, r^2^ = 0.99) was used to prepare the standard curve. The TPC of the samples was expressed as mg of gallic acid equivalents/g dry weight (g GAE/g DW) of extracts.

### 4.7. Measurement of Total Flavonoid Content

The aluminum chloride colorimetric method [73] was used to quantify the total flavonoid content in the methanolic extracts of *M. malabathricum* L. 0.02 mL of each extract was mixed with 0.06 mL of 70% methanol, 0.004 mL of 10% aluminum chloride, AlCl_3_ (AlCl_3_·6H_2_O), 0.004 mL of sodium acetate (NaC_2_H_3_O_2_·3H_2_O) (1 M), and 0.112 mL of distilled water. Subsequently, the absorbance was measured at 415 nm using a spectrophotometer (Multiskan^TM^ GO, Thermo Scientific, Waltham, MA, USA) after 40 min of incubation. The flavonoid concentrations were calculated by preparing a calibration curve using quercetin as the standard (ranging 0.10–0.55 mg/mL, r^2^ = 0.99). The flavonoid concentrations were expressed as quercetin equivalents in mg per gram of dry weight (mg/g DW) of extract. All assays were performed in triplicate.

### 4.8. DPPH (2,2-Diphenyl-1-Picrylhydrazyl) Radical Scavenging Activity Assay

DPPH free radical scavenging activity of the *M. malabathricum* L. methanolic extracts was analyzed using a microplate analytical assay following the standard procedure [73]. First, 0.05 mL of extracts at a series of concentrations (ranging 0.05–5.0 mg/mL) were added to 0.15 mL of DPPH solution (1 mM) in each well of a 96-well plate and incubated for 30 min at room temperature. A spectrophotometer (Multiskan^TM^ GO, Thermo Scientific, Waltham, MA, USA) was used to measure the absorbance at 515 nm. All extracts were assayed in triplicate and the data were used to determine the sample concentration required to scavenge 50% of the DPPH free radicals (IC_50_).

### 4.9. ABTS (2,2-Azinobis(3-Ethylbenzothiazoline-6-Sulfonic Acid)) Radical Scavenging Activity Assay

To determine the ABTS radical scavenging activities, a decolorization assay was used following Re, et al. [91] method. Firstly, 10 mL of 2.6 mM potassium persulfate was added with 10 mL of 7.4 mM ABTS solution and the mixture was incubated in the dark at room temperature. After 12 h, double distilled water (ddH_2_0) was used to dilute the mixture until the absorbance produced was 0.70 ± 0.2 at 734 nm. 0.02 mL of the extract samples of six different concentrations were mixed with 0.2 mL diluted ABTS^.+^ solution and incubated for 30 min at room temperature. Absorbance was taken in triplicates at the wavelength of 734 nm using a Multiskan^TM^ Go plate reader (Thermo Scientific, Waltham, MA, USA). The data were then obtained by calculating the sample concentration required to scavenge 50% of the ABTS free radicals (IC_50_).

(5) $${\mathrm{ABTS}}^{.+}\mathrm{scavenging}\;\mathrm{activity}\;\left(\%\right)=\frac{A_{0}-A_{1}}{A_{0}}\times 100$$

$${\mathrm{where}\;A}_{0}\;{=\mathrm{absorbance}\;\mathrm{of}\;\mathrm{ABTS}}^{.+}\mathrm{and}\;\mathrm{methanol}\;A_{1}\;{=\mathrm{absorbance}\;\mathrm{of}\;\mathrm{ABTS}}^{.+}\;\mathrm{and}\;\mathrm{sample}\;\mathrm{extract}\;\mathrm{or}\;\mathrm{standard}$$

### 4.10. Statistical Analysis

The data obtained in this study were subjected to statistical analysis using analysis of variance (ANOVA) and the mean values were compared using Tukey in the SPSS version 25 software. Pearson’s correlation analysis was also conducted to determine the relationship between the soil physicochemical characteristics with the bioactive phenolic, anthocyanin, and flavonoid present in the extracts, as well as the antioxidant properties.

## 5. Conclusions

This study has demonstrated that FW compost enhanced soil quality by increasing the soil pH, enhanced soil macromolecules, and improved micronutrients compared to the control. The study has also shown that FW compost enhanced the total chlorophyll and carotenoid contents. Furthermore, despite the addition of FW compost or PK biochar to acidic soil, both recorded a significant production of bioactive pigments and resulting better antioxidant activities in the leaf, stem, and root extracts. It was found that *M. malabathricum* L. ameliorate soil acidification by an increase in its soil macronutrients (total N, available P, K, Ca, and Mg), leaf pigment (chlorophylls content), secondary metabolites (TAC, TPC, and TFC) as well as DPPH and ABTS radical scavenging activities, while reducing soil micronutrients (Fe, Cu, Zn, Mn, Cd, and Al).

## Figures and Tables

**Table 1:** The effects of different treatments on the chemical properties of soil.

Treatments	Total N	Available P	Exchangeable Cation			
			K	Ca	Mg	Na
	%	mg/kg	meq/100g			
**T1**	3.33 × 10^−2^ ± 0.003 ^b^	2.500 ± 0.500 ^c^	15.3 × 10^−2^ ± 0.013 ^c^	1.287 ± 0.090 ^b^	28.7 × 10^−2^ ± 0.027 ^a^	3.03 × 10^−2^ ± 0.000 ^a^
**T2**	11.5 × 10^−2^ ± 0.005 ^a^	79.500 ± 0.500 ^b^	107 × 10^−2^ ± 0.130 ^b^	1.370 ± 0.100 ^b^	21.3 × 10^−2^ ± 0.015 ^a^	3.5 × 10^−2^ ± 0.005 ^a^
**T3**	12.3 × 10^−2^ ± 0.009 ^a^	190.500 ± 1.500 ^a^	170 × 10^−2^ ± 0.151 ^a^	2.280 ± 0.142 ^a^	27.7 × 10^−2^ ± 0.020 ^a^	4.0 × 10^−2^ ± 0.006 ^a^
**Treatments**	**Micronutrient**					
	**Zn**	**Fe**	**Mn**	**Cu**	**Cd**	**Al**
	**mg/kg**					
**T1**	2.653 ± 0.018 ^a^	191.270 ± 5.047 ^a^	2.653 ± 0.034 ^a^	31.090 ± 0.517 ^a^	79.3 × 10^−2^ ± 0.054 ^a^	2.1 × 10^−2^ ± 0.001 ^a^
**T2**	1.927 ± 0.070 ^c^	77.227 ± 1.998 ^c^	1.783 ± 0.107 ^c^	4.273 ± 0.069 ^b^	72.3 × 10^−2^ ± 0.049 ^a^	2.0 × 10^−2^ ± 0.000 ^a^
**T3**	2.140 ± 0.047 ^b^	133.747 ± 2.139 ^b^	2.340 ± 0.050 ^b^	4.613 ± 0.178 ^b^	67.0 × 10^−2^ ± 0.072 ^a^	1.9 × 10^−2^ ± 0.000 ^a^

Note: T1: control; T2: plant supplemented with palm kernel biochar; T3: plant; ppm: part per million; meq: milliequivalent. The values (mean ± SE) followed by dissimilar letters in each column are significantly different at *p* ≤ 0.05.

**Table 2:** Phytochemical screening of extracts from various parts of *M. malabathricum* L.

Phytochemical Screening	Leaves			Stem			Roots		
	T1	T2	T3	T1	T2	T3	T1	T2	T3
**Alkaloids I**	-	-	-	-	-	-	-	-	-
**Alkaloids II**	+	+	+	-	-	-	-	-	-
**Alkaloids III**	-	-	-	-	-	-	-	-	-
**Flavonoids I** **Flavonoid II**	++	++	++	+	+	+	+	++	++
	+++	+++	+++	+	+	++	+	++	++
**Phenol**	++	++	++	+	+	+	+	+	+
**Phlobatannins**	-	-	-	-	-	-	-	-	-
**Saponins**	-	-	-	-	-	-	-	-	-
**Tannins**	++	++	++	-	-	-	+	+	+

Notes: (+++) indicates appreciable amount of phytochemical; (++) indicate moderate amount of phytochemical; (+) indicate trace amount of phytochemical and (-) indicates the absence of the phytochemical.

**Table 3:** Comparison of chlorophylls and carotenoid contents from methanolic extract of *M. malabathricum* L. leaves.

	C*a* (µg g^−1^ DW)	C*b* (µg g^−1^ DW)	C*a* + C*b* (µg g^−1^ DW)	C*a*/C*b* Ratio	*C*(*x+c*) (µg g^−1^ DW)	C*a* + C*b*/*C*(*x+c*) Ratio
**T1**	182.623 ± 14.475 ^b^	90.628 ± 5.298 ^b^	273.252 ± 19.720 ^c^	2.010 ± 0.049 ^b^	149.212 ± 12.748 ^b^	1.836 ±0.028 ^a^
**T2**	261.837 ± 6.519 ^a^	95.008 ± 2.069 ^b^	356.845 ± 8.576 ^b^	2.756 ± 0.011 ^a^	224.698 ± 5.66 ^a^	1.588 ± 0.006 ^b^
**T3**	289.441 ± 8.881 ^a^	144.237 ± 4.585 ^a^	433.678 ± 13.224 ^a^	2.007 ± 0.025 ^b^	237.733 ± 7.224 ^a^	1.824 ± 0.014 ^a^

Note: T1: control; T2: plant supplemented with palm kernel biochar; T3: plant supplemented with food waste compost; C*a*: chlorophyll a; C*b*: chlorophyll b; C*a* + C*b*: total chlorophyll content; C*a*/C*b* ratio: Chlorophyll a and b ratio; *C*(*x+c*): carotenoid content; DW: dry weigh. The values (mean ± SE) followed by dissimilar letters in each column are significantly different at *p* ≤ 0.05.

**Table 4:** Effects of different treatments on total anthocyanin content, total flavonoid content, and total phenolic content in various parts of *M. malabathricum* L. methanolic extracts.

	TAC (mg/g DW)			TPC (mg GAE/g DE)			TFC (mg QE/g DE)		
	Leaves	Stem	Roots	Leaves	Stem	Roots	Leaves	Stem	Roots
**T1**	10.2 × 10^−2^ ± 0.036 ^b^	1.5 × 10^−2^ ± 0.005 ^a^	2.0 × 10^−2^ ± 0.006 ^a^	9505.160 ± 182.057 ^a^	2174.517 ± 27.789 ^b^	3606.996 ± 9.396 ^b^	1088.224 ± 31.536 ^b^	183.353 ± 1.264 ^b^	175.845 ± 4.395 ^c^
**T2**	26.5 × 10^−2^ ± 0.038 ^a^	6.1 × 10^−2^ ± 0.024 ^a^	6.5 × 10^−2^ ± 0.034 ^a^	9933.322 ± 30.217 ^a^	2267.808 ± 58.939 ^b^	5419.291 ± 36.121 ^a^	1524.796 ± 38.125 ^a^	183.572 ± 0.922 ^b^	643.268 ± 5.946 ^a^
**T3**	36.1 × 10^−2^ ± 0.034 ^a^	3.7 × 10^−2^ ± 0.015 ^a^	8.9 × 10^−2^ ± 0.020 ^a^	9857.329 ± 49.172 ^a^	4930.956 ± 16.025 ^a^	5396.671 ± 8.200 ^a^	1464.902 ± 16.032 ^a^	209.984 ± 0.572 ^a^	232.944 ± 2.511 ^b^

Note: T1: control; T2: plant supplemented with palm kernel biochar; T3: plant supplemented with food waste compost; TAC: total anthocyanin content; TPC: total phenolic content; TFC: total flavonoid content; DW: dry weight; GAE: gallic acid equivalent; QE: quercetin equivalent; DE: dry extract. The values (mean ± SE) followed by dissimilar letters in each column are significantly different at *p* ≤ 0.05.

**Table 5:** The effects of different treatments on the antioxidant activities of the various parts of *M. malabathricum* L. methanolic extracts.

	ABTS			DPPH		
	IC_50_ (mg/mL)			IC_50_ (mg/mL)		
	Leaves	Stem	Roots	Leaves	Stem	Roots
**T1**	46.4 × 10^−2^ ± 0.057 ^a^	450.7 × 10^−2^ ± 0.733 ^a^	133.5 × 10^−2^ ± 0.036 ^a^	22.3 × 10^−2^ ± 0.008 ^a^	376.9 × 10^−2^ ± 0.014 ^a^	299.9 × 10^−2^ ± 0.008 ^a^
**T2**	29.3 × 10^−2^ ± 0.027 ^b^	16.5 × 10^−2^ ± 0.002 ^b^	102.3 × 10^−2^ ± 0.061 ^b^	16.3 × 10^−2^ ± 0.002 ^b^	73.4 × 10^−2^ ± 0.039 ^c^	76.3 × 10^−2^ ± 0.035 ^b^
**T3**	27.9 × 10^−2^ ± 0.020 ^b^	19.7 × 10^−2^ ± 0.003 ^b^	14.8 × 10^−2^ ± 0.000 ^c^	13.1 × 10^−2^ ± 0.001 ^c^	276.6 × 10^−2^ ± 0.182 ^b^	49.1 × 10^−2^ ± 0.032 ^c^

Note: T1: control; T2: plant supplemented with palm kernel biochar; T3: plant supplemented with food waste compost; The values (mean ± SE) followed by dissimilar letters in each column are significantly different at *p* ≤ 0.05.

**Table 6:** Significant Pearson’s Correlation between Parameters.

	pH	N	P	K	Ca	Mg	Na	Zn	Fe	Mn	Cu	Cd	C*a*	C*b*	C*a* + C*b*	Car	TAC	TPC	TFC	DPPH	ABTS
pH	1																				
N	0.929 **	1																			
P	0.938 **	0.887 *	1																		
K	0.944 **	0.953 **	0.950 *	1																	
Ca	0.787 *	0.802 *	0.943 *	0.903 **	1																
Mg	−0.253	−0.40	0.115	−0.176	0.307	1															
Na	0.530	0.675	0.516	0.762 *	0.434	−0.249	1														
Zn	−0.802 **	−0.862 **	−0.628	−0.707 *	−0.459	0.490	−0.297	1													
Fe	−0.666	−0.814 *	−0.405	−0.614	−0.177	0.731 *	−0.353	0.939 **	1												
Mn	−0.515	−0.655	−0.233	−0.427	−0.115	0.575	−0.144	0.914 **	0.935 **	1											
Cu	−0.940 **	−0.975 **	−0.805	−0.907 **	−0.619	0.446	−0.549	0.938 **	0.868 **	0.751 *	1										
Cd	−0.547	−0.489	−0.952 **	−0.490	−0.351	0.415	−0.335	0.326	0.322	0.197	0.460	1									
C*a*	0.960 **	0.902 **	0.891 *	0.899 **	0.771 *	−0.343	0.494	−0.794 *	−0.691 *	−0.556	−0.912 **	−0.643	1								
C*b*	0.778 *	0.649	0.905 *	0.805 *	0.935 **	0.167	0.460	−0.290	−0.084	0.072	−0.533	−0.577	0.763 *	1							
C*a* + C*b*	0.950 **	0.851 **	0.968 **	0.904 **	0.862 **	−0.177	0.502	−0.657	−0.510	−0.360	−0.827 **	−0.657	0.972 **	0.894 **	1						
Car	0.945 **	0.914 **	0.829 *	0.883 **	0.720 *	−0.412	0.486	−0.843 **	−0.762 *	−0.639	−0.934 **	−0.622	0.994 **	0.687 *	0.940 **	1					
TAC	0.869 **	0.879 **	0.912 *	0.903 **	0.798 *	−0.107	0.548	−0.717 *	−0.573	−0.424	−0.843 **	−0.215	0.803 **	0.669 *	0.801 **	0.792 *	1				
TPC	0.675 *	0.767 *	0.548	0.616	0.340	−0.60	0.350	−0.748 *	−0.733 *	−0.670 *	−0.739 *	−0.490	0.702 *	0.360	0.618	0.727 *	0.442	1			
TFC	0.883 **	0.939 **	0.657	0.848 **	0.576	−0.524	0.514	−0.936 **	−0.902 **	−0.816 **	−0.967 **	−0.533	0.921 **	0.469	0.810 **	0.956 **	0.739 *	0.749 *	1		
DPPH	−0.979 **	−0.948 **	−0.943 **	−0.970 **	−0.817 *	0.240	−0.622	0.759 *	0.628	0.486	0.915 **	0.562	−0.954 **	−0.802 **	−0.954 **	−0.935 **	−0.840 **	−0.734 *	−0.867 **	1	
ABTS	−0.822 **	−0.785 *	−0.583	−0.779 *	−0.628	0.398	−0.464	0.745 *	0.713 *	0.585	0.836 **	0.582	−0.909 **	−0.575	−0.840 **	−0.925 **	−0.743 *	−0.460	−0.896 **	0.780 *	1

**. Correlation is significant at the 0.01 level (2-tailed); *. Correlation is significant at the 0.05 level (2-tailed).

**Table 7:** Physico-chemical properties of soil and organic fertilizers used in the study.

Properties	Soil	PK Biochar	FW Compost
pH	3.90	8.61	6.60
EC (dS/M)	0.10	3.67	2.84
Texture	Sandy loam	-	-
Total OC (%)	3.97	43.41	14.34
N (%)	0.06	0.5	2.39
Available P (mg/kg)	0.29	0.15	2.82
K (meq/100 g)	0.11	0.74	0.21
Ca (meq/100 g)	1.20	2.27	0.76
Mg (meq/100 g)	0.26	0.25	0.36

Note: EC: electrical conductivity; OC: organic carbon N: nitrogen; P: phosphorus; K: potassium; Ca: calcium; Mg: magnesium.

## Data Availability

Not applicable.
